# Bioaccumulation and Subchronic Toxicity of 14 nm Gold Nanoparticles in Rats

**DOI:** 10.3390/molecules21060763

**Published:** 2016-06-10

**Authors:** Clinton Rambanapasi, Jan Rijn Zeevaart, Hylton Buntting, Cornelius Bester, Deon Kotze, Rose Hayeshi, Anne Grobler

**Affiliations:** 1 DST/NWU Preclinical Drug Development Platform, Faculty of Health Sciences, North-West University, Potchefstroom 2531, South Africa; janrijn.zeevaart@necsa.co.za (J.R.Z.); 24861820@nwu.ac.za (H.B.); cor.bester@nwu.ac.za (C.B.); Rose.Hayeshi@nwu.ac.za (R.H.); Anne.Grobler@nwu.ac.za (A.G.); 2 Necsa, South African Nuclear Energy Corporation (SOC) Ltd., Pelindaba 2025, South Africa; deon.kotze@necsa.co.za

**Keywords:** gold nanoparticles, bioaccumulation, liver and kidney damage, Sprague Dawley rats

## Abstract

Colloidal suspensions of 14 nm gold nanoparticles (AuNPs) were repeatedly administered intravenously at three dose levels (0.9, 9 and 90 µg) to male Sprague Dawley rats weekly for 7 weeks, followed by a 14-day washout period. After sacrificing, the amount of gold was quantified in the liver, lungs, spleen, skeleton and carcass using neutron activation analysis (NAA). During the study, pre- and post (24 h) administration blood samples were collected from both the test and control groups, the latter which received an equal injection volume of normal saline. General health indicators were monitored together with markers of kidney and liver damage for acute and subchronic toxicity assessment. Histopathological assessments were done on the heart, kidneys, liver, lungs and spleen to assess any morphological changes as a result of the exposure to AuNPs. The mass measurements of all the groups showed a steady increase with no signs of overt toxicity. The liver had the highest amount of gold (µg) per gram of tissue after 56 days followed by the spleen, lungs, skeleton and carcass. Markers of kidney and liver damage showed similar trends between the pre and post samples within each group and across groups. The histopathological examination also showed no hepatotoxicity and nephrotoxicity. There was accumulation of Au in tissues after repeated dosing, albeit with no observable overt toxicity, kidney or liver damage.

## 1. Introduction

As described in various reviews and proof of concept studies gold nanoparticles (AuNPs) present a good strategy for drug and gene delivery as they can deliver a wide variety of cargoes [1,2,3,4]. In addition, a nanomedicine using AuNPs as a delivery vehicle has been assessed in a phase I pharmacokinetic study for cancer [5]. Several features of AuNPs make them well suited for drug delivery and the recent advances in the synthesis and characterization techniques of engineered nanomaterials (ENMs) [6] enable their biomedical applications to be exploited easily. However there is still no consensus [7,8] and a paucity of data with regards to the safety of AuNPs.

Risk is a function of the product of hazard (a material property), susceptibility of the organism and the exposure. Different models have been used to assess susceptibility [9,10], however, the bulk of toxicity assessment studies for AuNPs mainly varied the exposure by altering the nanomaterial properties. Nanomaterial properties that can be altered include *inter alia*; shape, size, surface area, surface charge and concentration. The influence of particle size on acute biodistribution and toxicity has been extensively studied [9,11,12,13,14,15], however, there is a dearth of information when it comes to longer term studies. Likewise, surface properties have been investigated mainly in short term studies [10,13,16,17,18]. From the published acute studies, there is a general agreement that liver and spleen uptake is high [14,18,19,20]. The influence of route of administration has been investigated in acute studies as well [21]. Considering what has been done, the information available is still insufficient to draw general conclusions due to differences in the study designs. This is not surprising considering that nanotoxicity is a fairly young discipline [22,23] with the principles of characterization of nanomaterials from the leading voices being only a decade old [24].

Bioaccumulation occurs when an organism takes up substances, in this case nanomaterials, at a rate higher than the clearance rate. The bioaccumulation propensity of any nanomaterial is dependent on its biopersistence in the organ/tissue. The route of exposure/administration has an influence on the organs that come into contact with the nanomaterial [24]. For systemic drug delivery purposes, using AuNPs the intravenous route is the most important to study since there is limited oral absorption [16]. The number of studies reporting on the biopersistence of AuNPs after intravenous administration are relatively few [19,25] and so are studies on their bioaccumulation [26,27]. The exposure an organ will have to a metal or nanomaterial will increase due to bioaccumulation, thus there is a clear need to have more information on the bioaccumulation of AuNPs after repeated intravenous administrations.

Safety assessments of AuNPs include end organ toxicity that can result from acute and/or subchronic exposure. The influence of bioaccumulation on end organ toxicity must be investigated to gather safety data. Serum enzymes and metabolites serve as good markers for hepatotoxicity and nephrotoxicity. In addition, histopathological examination is a good indicator to assess structural damage. This approach has previously been used in studies assessing the safety of AuNPs albeit with different results [27,28].

The aim of this study was to assess and quantify the bioaccumulation of Au in male Sprague Dawley rats after multiple intravenously administered doses of AuNPs. Little is known about the influence of dose (concentration of AuNPs) on the bioaccumulation of AuNPs after repeated administrations over weeks, thus different doses were used in the study. Levels of serum enzymes and metabolites were measured to assess if repeated dosing caused any kidney or liver damage. General health assessments were routinely done and histopathological assessment of tissues were done to assess for both overt and organ toxicity.

## 2. Results

### 2.1. Synthesis and Characterization AuNPs

**Figure 1:**
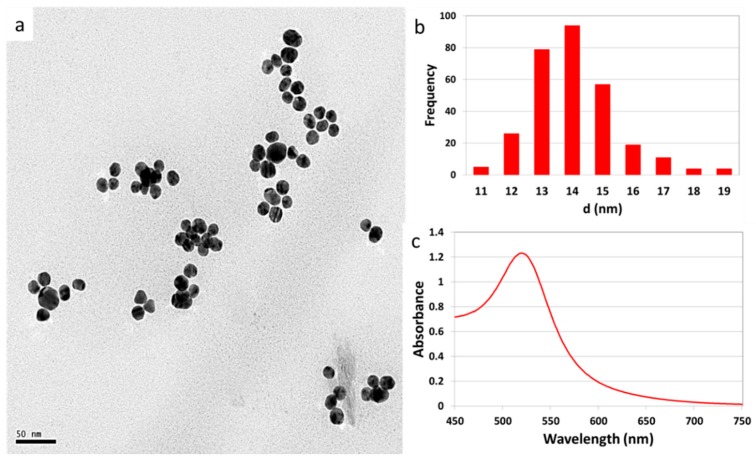
Characterization of AuNPs synthesized using the citrate reduction method. The (**a**) TEM image shows the morphology; (**b**) bar graph shows the size distribution and (**c**) UV/Vis spectra.

The citrate reduction method was used to synthesize 14 nm AuNPs using the Turkevich-Frens method [29,30]. The morphology and primary size (14 ± 1.2 nm) distribution as determined by TEM (see Figure 1) was as expected due to the careful selection of the ratio of the gold salt to the reducing agent.

The AuNPs had the characteristic resonance peak in the 520–530 nm wavelength range attributed to the 14 nm particles. The absence of secondary peaks confirmed the monodispersity of the colloidal suspension [31]. The zeta potential, which was negative because of the citrate surface coating and hydrodynamic size, is shown in Table 1 together with other dosimetric parameters.

### 2.2. Bioaccumulation of Au in Tissues after Repeated Dosing

**Figure 2:**
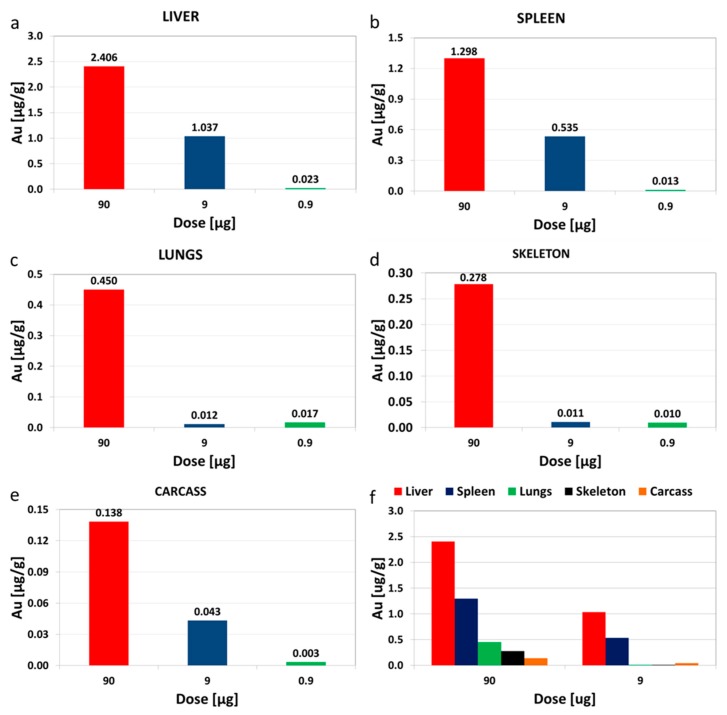
Bioaccumulation of Au in tissues. The rats received seven intravenous doses weekly at three dosing levels of 0.9, 9 and 90 µg. The Au was quantified using NAA in the LIVER (**a**); SPLEEN (**b**); LUNGS (**c**); SKELETON (**d**) and CARCASS (**e**) 2 weeks (washout period) after the last dose was administered. The bottom right graph (**f**) shows a comparison of the levels in the liver, spleen, lungs, skeleton and carcass at the 90 and 9 µg dose levels. Please note that the Y scale is different in all the organs with the liver having the most amount of Au per gram and the carcass having the least.

The amount of Au was determined in the liver, lungs, skeleton, spleen and carcass using neutron activation analysis (NAA). The organs were chosen based on results from an acute biodistribution study we conducted (data not shown). The liver and spleen had the highest amount of Au per gram of tissue across all dose levels used in the study (see Figure 2). The values are expressed as micrograms of Au per gram of organ.

Unlike in a previous report [27], the bioaccumulation in all tissues/organs did not exhibit any linear dose dependence patterns. There was no correlation between the amounts recovered in the organs and the dose administered. As a percentage of the total injected dose, the total recovery of Au at the end of the study was 17% (110 μg), 38% (24.2 μg) and 43% 2.7 μg) for the 90, 9 and 0.9 μg administered doses.

### 2.3. Toxicological Studies

**Figure 3:**
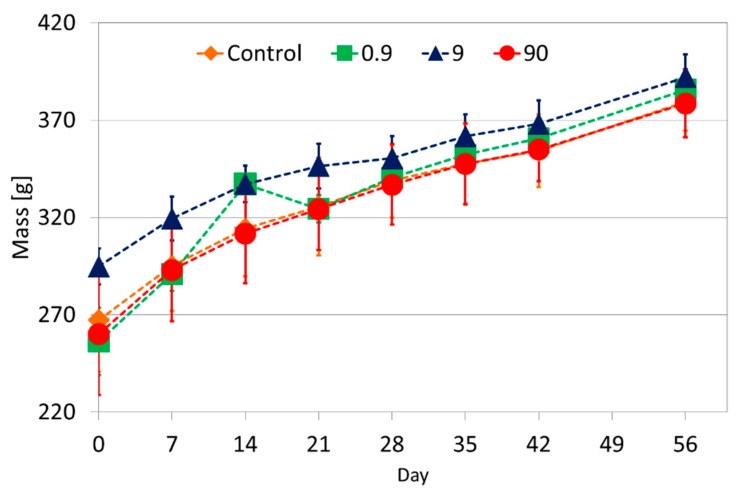
Comparison of weights of rats in the four treatment groups in the study over the 56 days. All the rats gained mass over time in a similar trend as expected for the species.

During the study all the physiological and behavioural indicators of the rats were monitored and the observations did not reveal any signs of overt toxicity. All injections were well tolerated, with none of the rats having to be sacrificed before the end of the study due to ethical considerations or distress. All the rats had a comparable steady weight gain (see Figure 3) with no differences between different groups being observed due to the exposure to AuNPs or the dose level.

### 2.4. Markers of Liver and Kidney Damage

**Figure 4:**
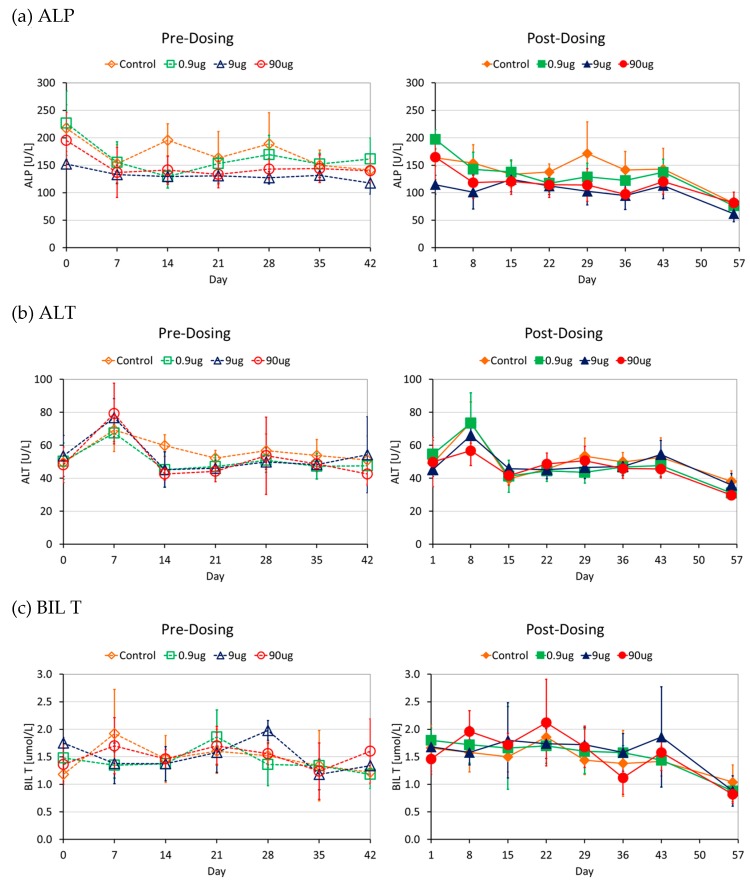
Similar trends were observed across all groups both for the pre- and post-dose measurements for markers of liver damage. The pre-dose measurements (on the left side) were done at baseline (T = 0) and 7 days after the preceding dose. The post dose measurements were done 1 day after dosing and the last one 14 days (washout period) after the last dose. ALP = Alkaline phosphatase (**a**); ALT = Alanine Phosphatase (**b**); and BIL T = Total Bilirubin (**c**).

The first pre-dose sample served as the baseline measurement and the other six samples were obtained before administration of the next dose which was also 7 days after the preceding dose. The post dose samples were obtained 1 day after each dose with the exception of the last one obtained 14 days after the last dose (washout period). The levels of ALT, ALP and BIL T which give an indication of liver damage showed similar trends in all groups in the study (see Figure 4).

**Figure 5:**
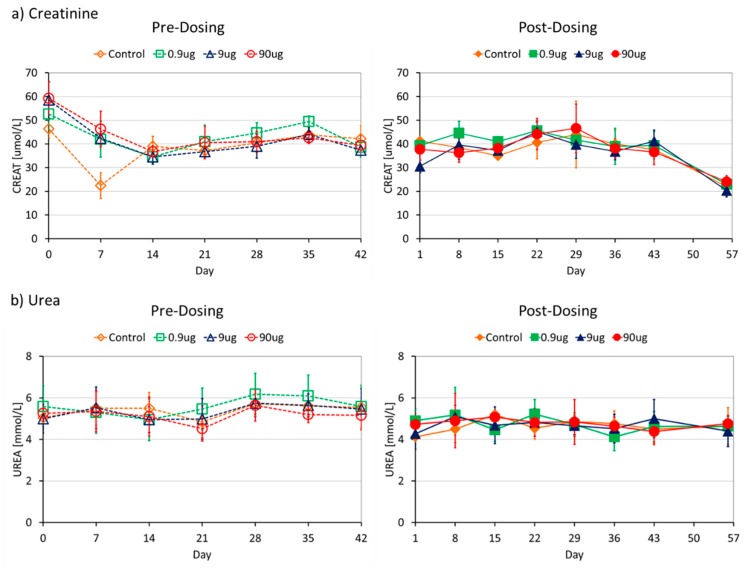
Similar trends were observed across all groups both for pre- and post-dosing measurements for the levels of markers of kidney damage (**a**) Creatinine and (**b**) Urea. The pre-dose measurements (on the left side) were done at baseline (T = 0) and 7 days after the preceding dose. The post dose measurements were done 1 day after dosing and the last one 14 days (washout period) after the last dose. Urea = Blood Nitrogen Urea, CREAT = Creatinine.

No differences were noticed between the pre- and post-dose levels. Analysis of the levels of creatinine and urea nitrogen, the metabolites that give an indication of kidney damage, showed similar trends in all the groups (see Figure 5). No differences were detected between the pre and post dose levels of these two metabolites.

### 2.5. Histopathology

**Figure 6:**
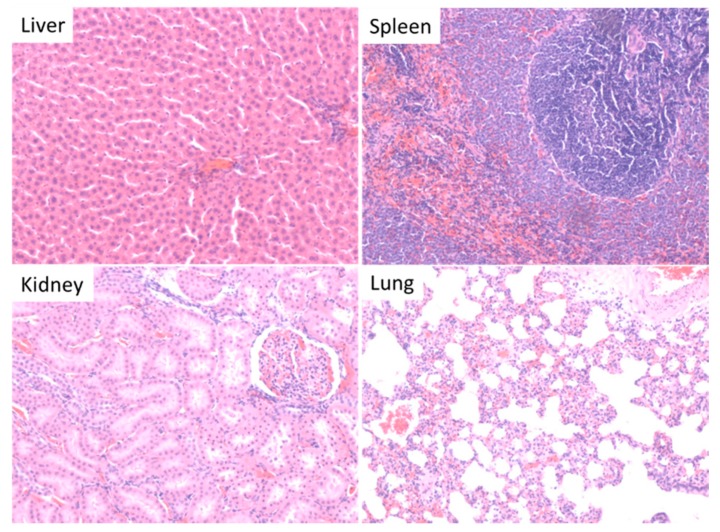
Representative histopathological images (×10 magnification) of the liver, spleen, kidneys and lungs after exposure to AuNPs. The organs represent all the treatment groups and the control as no abnormalities were detected in all the groups due to the exposure to AuNPs.

Exposure to AuNPs at the different dose levels did not result in any tissue damage as revealed by histopathological assessment of the; heart, kidneys, liver, lungs and spleen (see Figure 6). The assessment showed comparable results between all test groups and the control.

## 3. Discussion

Use of AuNPs for drug delivery can only be implemented successfully in the clinic when all questions with regards to the safety have been satisfactorily answered. The question on bioaccumulation of Au when administered as AuNPs for drug delivery purposes has not been extensively investigated and thus remains unanswered. There is a clear need for multifunctional nanocarriers (which AuNPs can be) with low bioaccumulation propensity [32]. Bioaccumulation is influenced by the biopersistence of a material which is a result of a biological system’s failure to clear foreign material. This study describes, the acute and subchronic toxicity and bioaccumulation of AuNPs after repeated dosing and a washout period in Sprague Dawley rats. The amount of Au was quantified using NAA and the toxicity endpoints were general health assessments and the monitoring of markers of kidney and liver damage in serum. The influence of dose on the bioaccumulation and acute and subchronic toxicity of AuNPs was investigated and no correlation was found when 14 nm size is used.

The bioaccumulation patterns followed the order liver > spleen > lungs > skeleton > carcass with the liver having the highest amount in micrograms per gram of the organ/tissue. Contrary to an earlier report [27], there was no proportional increase in the amount of Au in all the organs/tissues examined with an increase in the dose administered. It must be noted that in the earlier report the study duration was only 8 days. The amount of Au found in the organs was manifold higher than the background. The lowest amount detected (in all the samples analyzed) was a few times above the limit of detection of our quantification technique, this shows that all the Au was from the administered dose. A control group was not used in the study for quantifying Au because probability of finding natural Au in biological samples is low as described in literature [26]. Excretion of Au is mainly via the hepatobiliary system. Since rats are known to eat their feces, the amounts detected in tissues can only be attributed to the administered doses because little or no oral absorption was witnessed in our own study [33], which is also corroborated in the literature [16].

The percent recovery of the Au in our study is comparable to another published study [19] in which the authors recovered 32% of the injected dose. Due to the long duration of our study, it was not possible to use metabolic cages, hence, the collection of feces was not possible. The previously reported hepatobiliary excretion rate of 0.5% ID/day [13] might not be applicable in this study mainly because it was measured after a single dose whilst multiple doses were used in this study.

There is a paucity of data on the bioaccumulation of Au [26,27] and other engineered nanomaterials (ENMs) [34] in general. The studies reporting on bioaccumulation could not be easily generalized due to the vast differences in the doses, study designs and organs used to quantify the ENMs. However in all the studies, the liver and spleen had the highest accumulation levels indicating that the hepatobiliary system is the main clearance mechanism [14,19,25]. Just as for biodistribution, bioaccumulation is also thought to be influenced by surface properties of the ENMs [35].

Based on the general health assessments which focused on monitoring behavioural (cage side behaviour and feeding patterns) and physiological (monitoring of mass gain and alertness) indicators there were no differences between the control and test groups in this study. This was comparable to the results observed when the masses of rats were monitored when magnetite an ENM was administered at different doses in a rodent model [34]. The markers of liver damage, ALP, ALT and BIL T together with histopathological examination of liver tissues showed no differences between the control and tests groups. The same trend was observed for the markers of kidney damage; CREAT and UREA together with histopathological assessment of kidneys which is regarded as the gold standard for the assessment of nephrotoxicity [36]. Histopathological examination of the heart, lungs and spleen did not show any differences between the different groups. No influence of the dose level or the actual treatment with AuNPs was detected. The markers of liver and kidney damage were also similar between the pre and post dose samples indicating that there was no acute and/or subchronic damage.

Serum enzymes and metabolites are used as biomarkers for the assessment of drug induced liver injury (DILI) and nephrotoxicity, both acute and subchronic. If one considers the bioaccumulation levels of Au in the liver for instance, the lack of alteration in the levels of the markers assessed can be interpreted as a sign of safety or alternatively, that they are not the appropriate indicators with regards to nanomaterial safety. As with ENMs characterization techniques, there might be a need to come up with specific markers that can be used to assess nanomaterial induced liver injury (NILI) in the routine safety assessment of ENMs. The results of our study however are not in agreement with those of a previous study where they showed dose dependent detrimental effects on tissue histology changes [37]. The duration of this study was however short compared to ours but this highlights the lack of agreement on the issue of toxicity of AuNPs after repeated dosing.

### 3.1. Materials and Methods

#### 3.1.1. Preparation and Characterization of AuNPs

All chemicals used were of high purity or analytical grade. Spherical citrate coated 14 nm AuNPs were synthesized under sterile conditions using adapted modified Turkevich-Frens method [29,30]. Briefly 1 mM solution of hydrogen chloroauric acid (HAuCl_4_·3H_2_O) (Sigma, St. Louis, MO, USA) was brought to boil and 10 mL 38.8 mM of trisodium citrate was added until the solution turned wine red., the solution was refluxed for 30 min.

The average particle size and morphology of the colloidal suspensions were determined using transmission electron microscopy (TEM; FEI Tecnai G2, Eindhoven, The Netherlands). At least 250 particles were used to determine the primary size distributions using ImageJ software (version 1.48; National Institutes of Health, Bethesda, MD, USA). The UV-vis absorption spectra were obtained using a LAMBDA 1050 UV/Vis/NIR spectrophotometer (PerkinElmer, Waltham, MA, USA). Concentrations of the prepared AuNPs; molar, number and mass and surface area were calculated using size determined by TEM, mass of gold salt used and assumptions were made that the reaction goes to completion and the particles are spherical [38]. The hydrodynamic size and zeta potential were determined by dynamic light scattering (DLS) and electrophoretic potential, respectively, using a Zetasizer Nano (Malvern Instruments, Worcestershire, UK) at 25 °C. The average pH of the colloidal suspensions was 6.2.

#### 3.1.2. Animals and AuNPs Treatment

**Figure 7:**
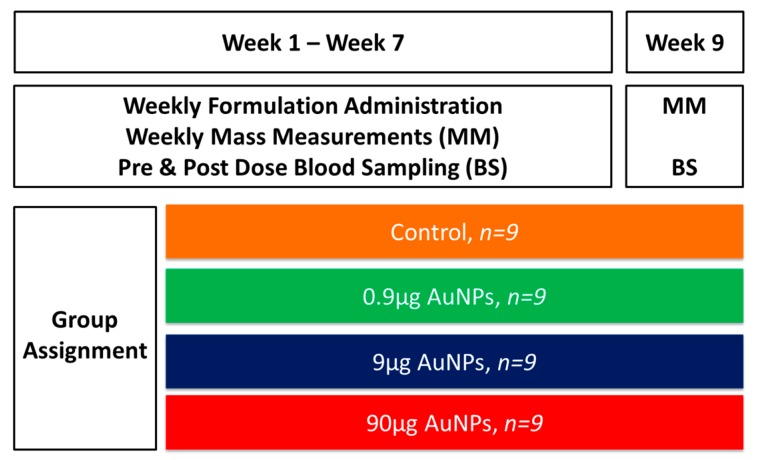
Study design for the bioaccumulation and subchronic toxicity of AuNPs.

Male Sprague Dawley rats, age: 8–10 weeks, weighing 240–300 g were used in the study. The rats were bred and procured from the Vivarium of the DST/NWU/Preclinical Drug Development Platform (Potchefstroom, South Africa). Animals were housed in stainless steel cages in groups of four under standard environmental conditions (22 ± 2 °C, 55% ± 15% RH) with access to water and food *ad libitum*. The study was conducted in accordance with the South African National Standard for the Care and Use of Animals for Scientific Purpose. Ethical approval was sought from and granted by the ethics committee of AnimCare of the North-West University (NWU-00029-14-A5). The rats were divided into 4 groups (*n* = 9) with each group receiving 90 µg, 9 µg, 0.9 µg or 0 µg of Au (mass concentration) in the form of AuNPs, intravenously with saline being used as the control (see Figure 7). The injection was administered once weekly for seven weeks via the tail vein and had a volume of 500 µL, which is within the acceptable range of intravenous injection in rats [39].

During the study, physiologic (mass gain over time) and behavioural (feeding habits and cage side behaviour) indicators were monitored [40] twice weekly to assess the general health. After the last dose there was a 14 d washout period before the termination of the study. On the last day of the study, all rats were sacrificed using an overdose of pentobarbitone (Euthapent^®^). In each group, the rats were further divided into the 2 groups; biodistribution group (*n* = 4) and the group used in the histopathological analysis to assess end organ toxicity (*n* = 5).

### 3.2. Determination of Au in Tissues: Neutron Activation Analysis

The liver, spleen, lungs, bones and remaining carcass of the rats in the biodistribution group were collected and weighed for the quantification of Au using neutron activation analysis (NAA) [26,41,42]. Briefly, all the samples were dried in an oven at 65 °C for 48 h, and then ashed at 650 °C over a 6 h period. All the bones were separated from the carcass. The final mass of the total ash was noted for each sample and approximately 200 mg of it weighed and placed in a trace element free polyethylene flip-top vial which were sealed by friction welding. The vials were then placed in larger vials making the sample doubly encapsulated. The samples were irradiated in a SAFARI-1 20 MW research reactor in a RINGAS pneumatic system for 8 s using a neutron flux of 10^14^ n·s^−1^·cm^−2^ triggering the ^197^Au (n, γ) ^198^Au nuclear reaction. Standards with 0.1, 0.5, 1 and 2 µg of Au in containers with the same geometry as the sample holders were run with the experimental samples as reference standards. The reference standards were used to calculate the amount of Au present in the samples. Blank samples were also irradiated for background correction. The γ decay energies of the samples were recorded using a well-type high purity germanium (HPGe) (NATS, Middletown, CT, USA) detector coupled to Genie 2000 program software. The 411 keV (95.6%) line was used to count the ^198^Au content and used to calculate the amount of Au in each sample.

### 3.3. Toxicological Studies

In the toxicity assessment group, pre- and post-dosing blood samples were collected in tubes with a clot activator and gel for serum separation (BD Vacutainer^®^) and immediately mixed as per manufacturer’s instructions. The tubes were centrifuged at 3000 *g* for 10 min and the prepared serum samples were stored at −80 C. Analysis to measure levels of alanine transferase (ALT), alkaline phosphatase (ALP) and total bilirubin (BIL T) was performed to assess liver damage. ALT and ALP are enzymes are found intracellularly or anchored on the cell membrane and are induced, leaked and/or shed from hepatocytes during liver injury [43,44]. Bilirubin, a normal product of *heme* catabolism, is excreted via conjugation in the liver and its levels in serum are increased in liver injury and inhibition of its conjugation and transport [45,46]. Kidney damage was assessed by measurement of the levels of creatinine (CREAT) and blood urea nitrogen (UREA). Both urea and creatinine are products of protein metabolism, which are cleared almost entirely by the kidneys. The serum levels of the enzymes and metabolites were done using the Cobas 6000 (Roche Diagnostics, Basel Switzerland). The kidneys, heart, lungs, liver and spleen were collected and fixed with 10% formalin. All samples were stored at room temperature until histopathological analysis was done by an independent laboratory (IDEXX, Pretoria, South Africa). Micrographic images were captured using an Olympus light microscope (Olympus, Tokyo, Japan).

### 3.4. Calculations and Statistical Analysis

The statistical significance of the differences between the mean values in the different groups was assessed using mixed linear models, which took into account repeated measures [47]. Statistical probability (*p*) values less than 0.05 were considered significantly different.

## 4. Conclusions

There was accumulation of Au in the order liver > spleen > lungs > skeleton > carcass per gram of tissue after seven weekly doses (three dose levels) and a 2-week washout period in Sprague-Dawley rats. There was no observable acute or subchronic toxicity that can be attributed to the use of AuNPs after the repeated dosing despite the accumulation in organs/tissues.

## Figures and Tables

**Table 1:** Dosimetry of AuNPs used in the study. Three dose levels were used with dosimetry expressed as mass and number concentrations and surface area of nanoparticles.

Parameter	Value		
Primary particle size	14 nm		
Hydrodynamic particle size	25 nm		
Zeta potential	−47 mV		
Administered mass of AuNPs (µg) per rat	90	9	0.9
Administered number of AuNPs per rat (10^12^)	3.3	0.33	0.033
Administered surface area (cm^2^)	20.2	2.02	0.202
